# Effects of obesity on bone metabolism

**DOI:** 10.1186/1749-799X-6-30

**Published:** 2011-06-15

**Authors:** Jay J Cao

**Affiliations:** 1 USDA ARS Grand Forks Human Nutrition Research Center 2420 2nd Ave N Grand Forks, ND 58202-9034, USA

**Keywords:** bone, fat, obesity, osteoporosis, inflammation

## Abstract

Obesity is traditionally viewed to be beneficial to bone health because of well-established positive effect of mechanical loading conferred by body weight on bone formation, despite being a risk factor for many other chronic health disorders. Although body mass has a positive effect on bone formation, whether the mass derived from an obesity condition or excessive fat accumulation is beneficial to bone remains controversial. The underline pathophysiological relationship between obesity and bone is complex and continues to be an active research area. Recent data from epidemiological and animal studies strongly support that fat accumulation is detrimental to bone mass. To our knowledge, obesity possibly affects bone metabolism through several mechanisms. Because both adipocytes and osteoblasts are derived from a common multipotential mesenchymal stem cell, obesity may increase adipocyte differentiation and fat accumulation while decrease osteoblast differentiation and bone formation. Obesity is associated with chronic inflammation. The increased circulating and tissue proinflammatory cytokines in obesity may promote osteoclast activity and bone resorption through modifying the receptor activator of NF-κB (RANK)/RANK ligand/osteoprotegerin pathway. Furthermore, the excessive secretion of leptin and/or decreased production of adiponectin by adipocytes in obesity may either directly affect bone formation or indirectly affect bone resorption through up-regulated proinflammatory cytokine production. Finally, high-fat intake may interfere with intestinal calcium absorption and therefore decrease calcium availability for bone formation. Unraveling the relationship between fat and bone metabolism at molecular level may help us to develop therapeutic agents to prevent or treat both obesity and osteoporosis.

Obesity, defined as having a body mass index ≥ 30 kg/m^2^, is a condition in which excessive body fat accumulates to a degree that adversely affects health [1]. The rates of obesity rates have doubled since 1980 [2] and as of 2007, 33% of men and 35% of women in the US are obese [3]. Obesity is positively associated to many chronic disorders such as hypertension, dyslipidemia, type 2 diabetes mellitus, coronary heart disease, and certain cancers [4-6]. It is estimated that the direct medical cost associated with obesity in the United States is ~$100 billion per year [7].

Bone mass and strength decrease during adulthood, especially in women after menopause [8]. These changes can culminate in osteoporosis, a disease characterized by low bone mass and microarchitectural deterioration resulting in increased bone fracture risk. It is estimated that there are about 10 million Americans over the age of 50 who have osteoporosis while another 34 million people are at risk of developing the disease [9]. In 2001, osteoporosis alone accounted for some $17 billion in direct annual healthcare expenditure.

Several lines of evidence suggest that obesity and bone metabolism are interrelated. First, both osteoblasts (bone forming cells) and adipocytes (energy storing cells) are derived from a common mesenchymal stem cell [10] and agents inhibiting adipogenesis stimulated osteoblast differentiation [11-13] and vice versa, those inhibiting osteoblastogenesis increased adipogenesis [14]. Second, decreased bone marrow osteoblastogenesis with aging is usually accompanied with increased marrow adipogenesis [15,16]. Third, chronic use of steroid hormone, such as glucocorticoid, results in obesity accompanied by rapid bone loss [17,18]. Fourth, both obesity and osteoporosis are associated with elevated oxidative stress and increased production of proinflammatory cytokines [19,20]. At present, the mechanisms for the effects of obesity on bone metabolism are not well defined and will be the focus of this review.

## Proinflammatory cytokines are elevated in obesity

Obesity is associated with low-grade chronic inflammation. The seminal finding that the expression of a proinflammatory cytokine, tumor necrosis factor-α (TNF-α), is elevated in the adipose tissue of obese mice provided the first evidence of a link between obesity and inflammation [21]. Later, the discovery of leptin, a small polypeptide hormone secreted primarily by the adipocytes, further supports that adipose is not just a energy storing organ and it is also an active endocrine tissue [22,23]. Since then, numerous experimental, epidemiological, and clinical studies have established that obesity is associated with a chronic inflammatory response, abnormal cytokine production, increased acute-phase reactants, and activation of inflammatory signaling pathways, and that these processes are involved in and responsible for the development of obesity-related diseases [24]. In obesity, adipose tissue is infiltrated with an increased amount of macrophages, which are an important source of inflammatory cytokines [25,26]. Obese humans express higher levels of TNF-α in adipose tissue than do lean individuals [27]. Adipose tissue also produces other proinflammatory factors including interlukin-6 (IL-6) and C-reactive protein (CRP) [28,29]. Obesity has also been implicated in the development or progression of musculoskeletal diseases such as osteoarthritis, a common inflammatory bone disease [30]. Numerous studies have confirmed that increased production of proinflammatory cytokines are critical in the development and progression of obesity-related health disorders [31].

Obese individuals show abnormal circulating levels of TNF-α, IL-6, CRP, adiponectin and leptin. Adiponectin and leptin, which also mediate chronic inflammation, are adipokines produced by adipose tissue. Leptin has pleiotropic effects that modulate energy expenditure, appetite, and neuroendocrine functions. Leptin, which is increased in obesity, has been found to stimulate inflammatory responses in humans [32,33]. In contrast adiponectin acts as an anti-inflammatory cytokine which suppresses TNF-α-induced NF-κB activation [34]. It has been found that plasma adiponectin concentrations are lower in obese subjects as compared to non-obese individuals [35].

In a cross-sectional study of 16,573 individuals in the third National Health and Nutrition Examination Survey (NHANES) (1984-1994), logistic regression analysis showed that odds ratios for an elevated serum CRP among individuals with a body mass index (BMI) of 25- < 30, 30- < 35, 35- < 40, and ≥40 were 1.51, 3.9, 6.11, and 9.30, respectively [36]. In another cross-sectional study, CRP, IL-6 and leptin were significantly positively related to degree of adiposity [37].

## Proinflammatory cytokines increase bone resorption

**Figure 1:**
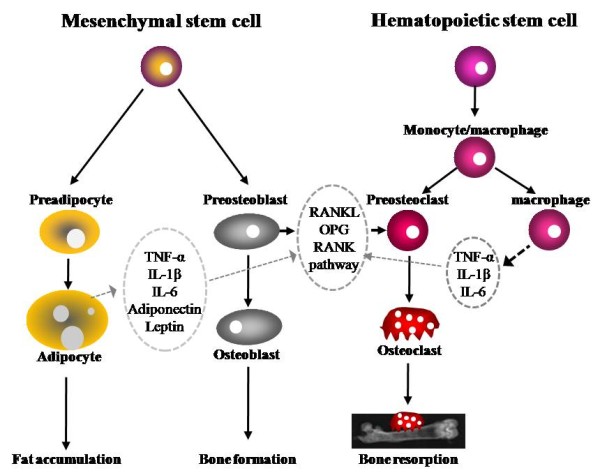
**Bone metabolism regulated by adipocytes, osteoblasts, and osteoclasts**. Fat accumulation is closely related to bone formation and resorption. Osteoblasts and adipocytes are derived from a common multipotential mesenchymal stem cell. Osteoclasts are differentiated from monocyte/macrophage precursors of hematopoietic stem cells origin. Adipocytes secrete several cytokines such as TNF-α, IL-1β, IL-6, adiponectin, and leptin which are capable of modulating osteoclastogenesis through RANKL/RANK/OPG pathway. IL, interleukin; OPG, osteoprotegerin; RANK, receptor activator of nuclear transcription factor κB; RANKL, receptor activator of nuclear transcription factor κB ligand; TNF-α, tumor necrosis factor alpha;

Bone is a dynamic organ that continuously undergoes significant turnover, a process called modeling/remodeling involving bone resorption by osteoclasts and bone formation by osteoblasts [38]. Therefore, bone mass at any particular time reflects the balance between bone formation and resorption. At the cellular level, osteoblast number and activity decrease while osteoclast number and activity increase with aging [39,40]. It is now established that osteoblasts regulate the recruitment and activity of osteoclasts through the expression of the receptor activator of NF-κB ligand (RANKL) and osteoprotegerin (OPG) (Figure 1). RANKL is expressed on the osteoblast/stromal cell surface and binds to its receptor, RANK, on the surface of hematopoietic precursor cells to stimulate osteoclast differentiation and maturation in the presence of macrophage colony stimulation factor (M-CSF). OPG, a decoy receptor secreted by osteoblasts, binds RANKL to prevent the activation of RANK and, therefore, to prevent osteoclast differentiation and activation [41,42]. It has been demonstrated that increased osteoclastic activity and increased bone resorption in postmenopausal women is positively correlated with the upregulation of RANKL [39,43,44].

Proinflammatory cytokines including TNF-α, IL-1, and IL-6 are key mediators in the process of osteoclast differentiation and bone resorption. Chronic inflammation and increased proinflammatory cytokines induce bone resorption and bone loss in patients with periodontitis [45], pancreatitis [46], inflammatory bowel disease [47], and rheumatoid arthritis [48]. It has also been established that upregulated proinflammatory cytokines are primary mediators of osteopenia or osteoporosis. The accelerated bone loss at menopause is linked to increased production of proinflammatory cytokines including TNF-α, IL-1, and IL-6 [20]. These proinflammatory cytokines are capable of stimulating osteoclast activity through the regulation of the RANKL/RANK/OPG pathway [49,50]. In mice lacking IL-1β and TNF genes [51] or over-expressing soluble TNF-α decoy receptor [52], ovariectomy did not cause bone loss. Blocking the action of IL-1 with an IL-1 receptor antagonist, or the signaling of TNF-α with a TNF-binding protein, decreased osteoclast formation and bone resorption in ovariectomized mice [53]. The significant increase in the development of osteoarthritis in obese human subjects is another evidence that chronic inflammation influences bone metabolism [30].

## Obesity affects bone turnover

Obesity is traditionally thought to be beneficial to bone and, thus, to protect against osteoporosis [5,54,55]. Mechanical loading stimulates bone formation by decreasing apoptosis and increasing proliferation and differentiation of osteoblasts and osteocytes [56] through the Wnt/β-catenin signaling pathway [57,58]. Therefore, mechanical loading conferred by body weight is part of the assumption that has led to widespread belief that obesity may prevent bone loss and osteoporosis [59-63].

However, recent reports have shown that excessive fat mass may *not *protect humans from osteoporosis and in fact, increased fat mass is associated with low total bone mineral density and total bone mineral content [64-67]. In a cross-sectional study of 60 females between 10 and 19 years of age, the percent of body fat was linked to suboptimal attainment of peak bone mass [68]. Increased adiposity may also be linked to the increased risk of bone fracture. For example, in a case-control study of 100 patients with fractures and 100 age-matched fracture-free control subjects aged 3 to 19 years, high adiposity are associated with increased risk of distal forearm fractures [69]. In another large cross-sectional study of about 13,000 adult men, pre- and post-menopausal women, percentage of body fat was positive associated with osteopenia and nonspine fractures [66].

In a leptin-deficient (ob/ob) mouse model for obesity, mice weighed twice as much as lean mice but had lower femoral bone mineral density, cortical thickness, and trabecular bone volume [70]. Obviously the positive effect of mechanical loading of increased body weight could not overcome the detrimental effect of leptin-deficiency (or possibly obesity) on bone in these mice. The apparent competing effects of adiposity and mechanical loading on bone metabolism remain an active research area. Research findings suggest that factors other than body weight are involved in the final outcome of obesity on bone health.

While research with obese animal model has established the negative effects of adiposity on bone metabolism, studies with human subjects continue to be controversial. Human obesity is a complex issue which in general involves excessive consumption of other nutrients, such as protein and minerals, known to influence bone metabolism [71]. Findings of the effects of obesity on bone health in humans have been based on statistical correlation or modeling rather than controlled trials. Thus, controlled studies with the obese animal model are useful for dissecting the mechanisms upon which excessive fat accumulation affect on bone metabolism.

Using a diet-induced obese mouse model, we demonstrated that feeding mice a high-fat diet (45% energy as fat) for 14 wks decreases trabecular bone volume and trabecular number in the proximal tibia despite a substantial increase in body weight and bone formation markers in cultured BMSC [72]. These structural changes are accompanied by increases in serum leptin and TRAP levels, the ratio of RANKL/OPG expression in cultured osteoblasts, and the number of TRAP-positive osteoclasts [72,73]. Increased osteoclast activity and decreased expression of IL-10, an anti-inflammatory cytokine, by bone marrow-derived macrophages in diet-induced obese mice have also been reported by others [74]. High fat-induced obese animals exhibited increased bone marrow adiposity accompanied by reduced BMD in different skeletal sites, up-regulation of peroxisome proliferator-activated receptor γ, cathepsin k, IL-6 and TNF-α [75].

Based on available literature, obesity appears to affect bone metabolism through several mechanisms. Obesity may decrease bone formation (osteoblastogenesis) while increasing adipogenesis because adipocyte and osteoblasts are derived from a common multi-potential mesenchymal stem cell (Figure 1) [76]. For example, mechanical loading promotes osteoblast differentiation and inhibits adipogenesis by down-regulating peroxisome proliferator-activated receptor gamma (PPARγ) or by stimulating a durable beta-catemin signal [12,13]. Activation of PPARγ by thiazolidinediones decreased osteoblast differentiation, bone mineral density and trabecular bone mass while increasing adipocytes differentiation and bone marrow adipose tissue volume [11,77,78].

Obesity may increase bone resorption through upregulating proinflammatory cytokines such as IL-6 and TNF-α. These proinflammatory cytokines are capable of stimulating osteoclast activity through the regulation of the RANKL/RANK/OPG pathway [49,50]. Obesity is significantly associated with degenerative and inflammatory musculoskeletal system [79]. Bone marrow adipocytes also may directly regulate the osteoclast progenitors, hematopoietic cells [80]. For example, when expressed with a dominant-negative form of CCAAT-enhancer-binding proteins (C/EBP) under the adipocyte fatty-acid-binding protein 4 promoter, mice cannot form adipocytes [81]. These mice lack white adipose tissue and have increased bone mineral density [82].

Obesity may affect bone metabolism directly or indirectly through adipocyte-derived cytokines such as leptin and adiponectin. Obesity is associated with significant increase in serum leptin [32,33] and decrease in adiponectin [35]. The action of leptin on bone appears to be complex and both positive [83,84] and negative [85,86] effects have been reported. It appears that its action may depend on current leptin status and the mode of the action (central or peripheral effects). Overproduction of leptin, as seen in obese animal models, may have negative effects on bone metabolism [73]. Increased serum leptin level has been found a negative regulator of bone mass in a mouse model [85]. Adiponectin is another cytokine secreted by adipocytes and has anti-inflammatory effect [34]. In animal model, adiponectin has been reported to inhibit osteoclastogenesis, reduce bone resorption, and increase bone mass [87]. Obese subjects have low serum adiponectin concentrations as compared to those normal subjects [35]. Increased secretion of leptin (and/or decreased production of adiponectin) by adipocytes may also contribute to macrophage accumulation by simulating transport of macrophages to adipose tissue [88] and promoting adhesion of macrophages to endothelial cells, respectively [89].

Finally, a high-fat diet, often a cause of obesity, has been reported to interfere with intestinal calcium absorption. Free fatty acids can form unabsorbable insoluble calcium soaps and therefore contributing to low calcium absorption [90-92].

Increased body weight associated with obesity may counteract the detrimental effects of obesity on bone metabolism. It is well established that body weight or body mass index (BMI) is positively correlated with bone mineral density or bone mass [59,93] and low body weight or BMI is a risk factor for low bone mass and increased bone loss in humans [60]. However, studies indicate the positive effects of body weight could not completely offset the detrimental effects of obesity on bone, at least in obese animal models.

## Conclusions

Accumulating data suggest that obesity is detrimental to bone health despite potential positive effects of mechanical loading conferred by increased body weight with obesity on bones. The decreased bone mass with obesity may be due to increased marrow adipogenesis at the expense of osteoblastogenesis, and/or increased osteoclastogenesis because of up-regulated production of proinflammatory cytokines, and/or excessive leptin secretion, or reduced adiponectin production, and/or reduced calcium absorption associated with high fat intake. Understanding the relationship between obesity and bone metabolism may help identify new molecular targets that can increase osteoblastogenesis while inhibiting adipogenesis and/or decreasing osteoclastogenesis. Ultimately, this knowledge may lead us to develop new therapeutic interventions to prevent both obesity and osteoporosis.

## List of abbreviations

CRP: C-reactive protein; IL: interleukin; OPG: osteoprotegerin; RANK: receptor activator of nuclear transcription factor κB; RANKL: receptor activator of nuclear transcription factor κB ligand; TNF-α: tumor necrosis factor alpha; TRAP: tartrate-resistant acid phosphatase; BMI: body mass index;

## Conflict of interests

The authors declare that they have no competing interests.

## Author's information

Dr. Cao received a Doctoral degree in nutrition from the University of Florida, Gainesville, Florida, USA. He worked as a postdoctoral research fellow in mineral nutrition at the Food Science and Human Nutrition Department, University of Florida and in bone biology at the Department of Medicine, University of California at San Francisco. Dr. Cao has published more than 30 papers in nutrition and bone biology fields. He has presented his research at many national and international conferences. Currently, he is a Research Nutritionist at the USDA ARS Grand Forks Human Nutrition Research Center where he conducts research focusing on the nutritional and physical activity regulation of bone metabolism using obese animal models. Dr. Cao also investigates the effects of dietary protein and acid-base balance on calcium absorption, retention, and markers of bone metabolism in human subjects.
